# From Conception to Delivery: A Comprehensive Review of Thyroid Disorders and Their Far-Reaching Impact on Feto-Maternal Health

**DOI:** 10.7759/cureus.53362

**Published:** 2024-02-01

**Authors:** Jalormy S Joshi, Amardeep Shanoo, Nainita Patel, Aishwarya Gupta

**Affiliations:** 1 Obstetrics and Gynaecology, Jawaharlal Nehru Medical College, Datta Meghe Institute of Higher Education and Research, Wardha, IND

**Keywords:** postpartum considerations, intervention, screening, feto-maternal health, pregnancy, thyroid disorders

## Abstract

This comprehensive review delves into the multifaceted landscape of thyroid disorders during pregnancy, exploring their impact from conception to postpartum considerations. Key findings highlight the intricate interplay between maternal thyroid health and fetal development, emphasizing the critical importance of timely screening and targeted interventions. The evolving landscape of research and technology suggests a paradigm shift toward personalized approaches in clinical practice, emphasizing integrated care models and the integration of telehealth platforms. Postpartum considerations, including postpartum thyroiditis, underscore the necessity for ongoing monitoring and intervention for maternal well-being. Implications for clinical practice encompass healthcare provider education, public awareness campaigns, and policy advocacy for standardized screening guidelines. The call to action resonates for increased research funding to advance understanding and improve outcomes. By fostering awareness, education, and collaborative efforts, this review aims to navigate the complexities of thyroid disorders during pregnancy, ensuring a healthier start for both mothers and their infants.

## Introduction and background

The intricate dance of hormonal balance during pregnancy plays a pivotal role in the well-being of both the mother and the developing fetus. Among the myriad factors influencing this delicate equilibrium, the thyroid gland emerges as a key orchestrator, regulating critical physiological processes [1]. It is imperative to elucidate the intricate nature of thyroid disorders to lay the foundation. The thyroid gland, a small butterfly-shaped organ in the neck, produces hormones crucial for metabolism, growth, and energy regulation. When this finely tuned system experiences disruptions, thyroid disorders emerge, encompassing conditions such as hyperthyroidism, hypothyroidism, and autoimmune disorders like Hashimoto's thyroiditis and Graves' disease. Understanding the spectrum of thyroid disorders is fundamental to grasping their multifaceted impact on pregnancy [2].

The significance of thyroid health in pregnancy cannot be overstated. The thyroid hormones thyroxine (T4) and triiodothyronine (T3) influence crucial aspects of fetal development, including brain and nervous system formation. Furthermore, thyroid function extends to maternal cardiovascular health, energy metabolism, and the intricate balance of hormones necessary for a successful pregnancy. A delicate interplay between the mother's thyroid and the developing fetus is thus woven, with deviations from the norm potentially leading to far-reaching consequences [3].

This review endeavors to synthesize existing knowledge and research, offering a panoramic view of thyroid disorders' various facets and their implications for feto-maternal health. By delving into the intricacies of thyroid function from the earliest stages of conception to delivery, the aim is to provide a resource that educates and sparks awareness among healthcare professionals, researchers, and the wider community. Through a nuanced exploration of diagnosis, management, and outcomes, this review seeks to contribute to the ongoing dialogue surrounding thyroid health in pregnancy, fostering a more profound understanding that may pave the way for enhanced clinical practices and improved maternal and fetal outcomes.

## Review

Prevalence of thyroid disorders in pregnancy

Epidemiology and Statistics

According to several studies, the prevalence of thyroid disorders in pregnancy varies between 10.4% and 33.9%, with hypothyroidism being more common than hyperthyroidism. The incidence of hypothyroidism in pregnancy is between 0.5% and 3.5%, while hyperthyroidism occurs in 0.2% to 0.4% of pregnant women and is most commonly associated with Graves' disease. Maternal thyroid dysfunction during pregnancy may lead to adverse outcomes such as preterm delivery, preeclampsia, miscarriage, low birth weight, and impaired neuropsychological development in the offspring. Early diagnosis and treatment of thyroid disorders before and during pregnancy are essential for the health of both the mother and the baby [3-9].

Risk Factors for Thyroid Disorders in Pregnant Women

The prevalence of thyroid disorders in pregnancy varies, with hypothyroidism being more common than hyperthyroidism. The overall prevalence of thyroid disorders in pregnancy has been reported as 10.4% to 33.9%, with hypothyroidism ranging from 31.6% to 13.2% and hyperthyroidism from 2.3% to 2.8% in different studies [3,4,7]. Several risk factors for thyroid disorders in pregnant women have been identified, including a history of thyroid disease, type 1 diabetes mellitus, other autoimmune diseases, current or past use of thyroid therapy, and a family history of autoimmune thyroid disease [5]. It is essential to screen pregnant women at high risk for thyroid disease to ensure early diagnosis and appropriate management, as maternal thyroid dysfunction can have significant implications for both the mother and the baby [10].

Effects of thyroid disorders on conception

Impact on Fertility

Thyroid disorders exert a substantial influence on fertility, creating a complex interplay between hormonal imbalances and reproductive function. In the case of hypothyroidism, insufficient production of thyroid hormones can disrupt the menstrual cycle, leading to irregular ovulation and anovulation. This, in turn, can pose challenges for couples attempting to conceive, prolonging the time it takes to achieve pregnancy [11]. Conversely, hyperthyroidism can also pose hurdles to fertility. The overproduction of thyroid hormones may result in menstrual irregularities and, in more severe cases, amenorrhea. Additionally, hyperthyroidism can impact the delicate hormonal balance required for successful implantation and the early stages of embryonic development [12]. Understanding and addressing the impact of thyroid disorders on fertility is paramount for couples seeking to conceive. Thyroid function assessments should be an integral part of fertility evaluations, enabling timely interventions and personalized treatment plans to optimize the chances of successful conception [13].

Thyroid Disorders and Reproductive Technologies

In the landscape of assisted reproductive technologies (ART), where precise timing and hormonal balance are critical, the role of thyroid disorders becomes even more pronounced. Both hypothyroidism and hyperthyroidism can affect the success rates of fertility treatments such as in vitro fertilization (IVF) and intrauterine insemination [14]. In the context of IVF, women with untreated thyroid disorders may experience lower implantation rates and an increased risk of miscarriage. Therefore, pre-conceptional screening for thyroid function and subsequent management become integral components of optimizing outcomes in ART procedures [15].

Moreover, the impact of thyroid disorders extends beyond the female partner. Research suggests that suboptimal thyroid function in men may also be associated with reduced fertility, emphasizing the importance of a comprehensive approach when evaluating couples undergoing reproductive technologies [14]. As advancements in reproductive medicine continue, recognizing and addressing the intricate relationship between thyroid health and assisted reproduction becomes paramount. A collaborative effort between reproductive endocrinologists, fertility specialists, and endocrinologists is essential to tailor interventions that enhance the success of reproductive technologies for couples grappling with thyroid disorders and fertility challenges [16].

Thyroid disorders in the first trimester

Normal Thyroid Function in Early Pregnancy

Intricate hormonal adaptations mark the first trimester to support the developing embryo. Thyroid hormones play a central role in this process, with the thyroid gland increasing in size and producing higher levels of thyroxine (T4) and triiodothyronine (T3). This surge in thyroid hormone production is essential for the early stages of fetal neurodevelopment and growth [17]. Maternal thyroid function undergoes subtle modifications as well. Thyroid-stimulating hormone (TSH) levels tend to decrease due to the stimulatory effect of human chorionic gonadotropin (hCG), a hormone secreted by the developing placenta. While these alterations are part of the normal physiological response to pregnancy, any deviations from the expected patterns may warrant careful consideration and evaluation [18].

Hyperthyroidism and Hypothyroidism in the First Trimester

Hyperthyroidism: Hyperthyroidism, prevalent during the first trimester, is often intricately linked to autoimmune disorders, with Graves' disease being a prominent example. In this condition, the immune system mistakenly attacks the thyroid, leading to an overproduction of thyroid hormones. Elevated levels of these hormones present significant challenges during pregnancy, contributing to a heightened risk of complications. Among these complications, gestational hypertension, preterm birth, and low birth weight are notable concerns. Furthermore, untreated hyperthyroidism can escalate into a rare yet severe condition known as a thyroid storm, demanding immediate and intensive medical attention. Managing hyperthyroidism in pregnancy requires a delicate balance to mitigate adverse outcomes for both the mother and the developing fetus [19].

Hypothyroidism: Hypothyroidism, characterized by an underactive thyroid gland, can also manifest in the initial trimester of pregnancy. In this condition, the thyroid produces insufficient thyroid hormones, posing potential risks to fetal neurodevelopment and overall growth. The consequences extend beyond the fetal realm, as the mother faces an elevated risk of complications, including gestational hypertension, preeclampsia, and gestational diabetes. Additionally, untreated hypothyroidism heightens the likelihood of adverse pregnancy outcomes, including miscarriage and preterm birth. The management of hypothyroidism during pregnancy necessitates vigilant monitoring and tailored interventions to ensure adequate thyroid hormone levels for optimal maternal and fetal well-being. Addressing these thyroid disorders in the first trimester is critical for minimizing associated risks and fostering a healthier pregnancy journey [20].

Risks and Complications

Thyroid disorders in the first trimester of pregnancy can have significant risks and complications for both the mother and the baby. Uncontrolled hypothyroidism during pregnancy can lead to various adverse outcomes, including preeclampsia, anemia, abruption, miscarriage, low birth weight, stillbirth, and, rarely, congestive heart failure [10]. Additionally, untreated thyroid disorders during pregnancy may lead to premature birth, low birth weight, developmental issues, and other serious complications such as preeclampsia and miscarriage [21-23]. Maternal hypothyroidism has been associated with an increased risk of low birth weight, fetal distress, and impaired neurodevelopment [24]. Pregnant women with thyroid disorders need to receive medical attention and appropriate management, especially during the first trimester, to mitigate these risks and complications [21].

Thyroid disorders in the second and third trimesters

Changes in Thyroid Function During Pregnancy

Thyroid disorders in the second and third trimesters of pregnancy can still pose risks and complications, although the prevalence of thyroid dysfunction is lower than in the first trimester [4]. The thyroid function during pregnancy changes due to the complex hormonal changes during this period [24]. Thyroid hormone requirements are higher in pregnancy, and thyroid hormone levels can be affected by hCG and increased thyroid hormone metabolism [24]. Thyroid dysfunction during pregnancy can lead to the development of preeclampsia, a condition characterized by high blood pressure and proteinuria [10]. Maternal thyroid dysfunction can result in preterm delivery, which can lead to various complications for both the mother and the baby [21]. Thyroid disorders during pregnancy can lead to low birth weight, which can be associated with various health issues for the newborn [8]. Maternal thyroid dysfunction can increase the risk of neonatal morbidity, affecting the health of the newborn [10]. Healthcare providers must monitor thyroid function during the second and third trimesters of pregnancy and provide appropriate management to minimize the risks and complications associated with thyroid disorders [4,24].

Gestational Thyroid Disorders

Gestational hyperthyroidism: Gestational hyperthyroidism takes center stage predominantly in the second and third trimesters of pregnancy, marked by a transient surge in thyroid activity often linked to increased hCG levels. While this condition typically does not impose significant risks on the developing fetus, severe cases warrant careful attention due to potential complications. Complications may include the onset of preeclampsia, a condition characterized by high blood pressure and organ damage, as well as an increased likelihood of preterm birth. Managing gestational hyperthyroidism necessitates meticulous monitoring and tailored interventions, often involving anti-thyroid medications. Striking a delicate balance in treatment is crucial to prevent adverse outcomes and ensure the well-being of both the expectant mother and the fetus [25].

Gestational hypothyroidism: Gestational hypothyroidism, characterized by an underactive thyroid during pregnancy, poses significant implications for fetal neurodevelopment and overall growth. Left untreated, this condition elevates the risk of several adverse outcomes, including preeclampsia, gestational diabetes, and preterm birth. Identifying and managing gestational hypothyroidism is paramount, typically involving thyroid hormone replacement therapy. Ensuring optimal maternal and fetal health requires a proactive approach to maintaining thyroid hormone levels within the recommended range. By addressing gestational hypothyroidism through timely interventions, healthcare providers can mitigate potential risks and contribute to a healthier pregnancy journey for both the mother and the developing fetus [10].

Maternal and Fetal Complications

Thyroid disorders in the second and third trimesters of pregnancy can lead to various maternal and fetal complications. Uncontrolled hypothyroidism during pregnancy has been associated with conditions such as preeclampsia, anemia, abruption, miscarriage, low birth weight, stillbirth, and, rarely, congestive heart failure [10]. Maternal thyroid dysfunction is also linked to an increased risk for early abortion, preterm delivery, neonatal morbidity, and other obstetrical complications [8]. Additionally, thyroid dysfunctions such as hypothyroidism, thyrotoxicosis, and thyroid nodules may lead to adverse outcomes such as placental abruptions, preeclampsia, preterm delivery, and reduced intellectual function in the offspring [10]. Pregnant women with thyroid disorders need to receive medical attention and appropriate management to minimize these risks and complications, especially during the second and third trimesters of pregnancy [8,10].

Diagnosis of thyroid disorders during pregnancy

S*creening Guidelines*

Universal vs. high-risk screening: Recent consensus guidelines do not recommend universal thyroid function screening during pregnancy but suggest testing high-risk pregnant women with a personal history of thyroid or other autoimmune disorders or with a family history of thyroid disorders [26,27]. Although universal screening may fulfill most criteria for a beneficial and cost-effective approach, areas of uncertainty remain, especially regarding the significance of borderline biochemical results [28]. Different medical societies do not recommend a universal screening approach early during pregnancy, but the topic remains a recurrent question in the literature [29]. Therefore, the current evidence and guidelines support targeted screening of high-risk pregnant women for thyroid function abnormalities rather than universal screening for all pregnant women.

Timing of screening: The timing of newborn screening (NBS) for thyroid disorders varies, but it typically involves the collection of a blood sample from the infant. The second screen for blood spot disorders, including thyroid screening, is usually recommended to be collected between five and 10 days of life, although the statewide average can be around 15.8 days. This timing is essential to ensure the early detection and treatment of certain rare diseases that can have severe implications if not identified and addressed promptly [30,31]. The first hearing screening for newborns typically occurs within one to two days of birth [32]. The blood test results from NBS are usually ready when the baby is five to seven days old [30]. Therefore, the timing of NBS for thyroid disorders and other conditions is designed to enable early detection and intervention, which can significantly impact the long-term health outcomes for the infant.

Diagnostic Tests and Interpretation

Thyroid function tests: Thyroid function tests diagnose thyroid disorders during pregnancy. Accurate assessment of thyroid function during pregnancy is critical for initiating thyroid hormone therapy and adjusting thyroid hormone doses in those already receiving thyroid hormone [33]. Trimester-specific reference intervals for thyroid function tests are critical for maintaining the delicate balance of thyroid hormones during pregnancy [33]. TSH and FT4 testing usually confirm the diagnosis of hyperthyroidism, and patients with Graves' disease usually have positive test results for thyroid-stimulating immunoglobulins [33]. Symptoms of hyperthyroidism may mimic those of normal pregnancy, such as an increased heart rate, sensitivity to hot temperatures, and fatigue [21]. Universal thyroid screening in pregnancy is a critical debate in thyroidology and obstetrics. While it fulfills most criteria for a beneficial and cost-effective approach, areas of uncertainty remain, especially regarding the significance of borderline biochemical results [28]. Therefore, targeted screening of high-risk pregnant women for thyroid function abnormalities is currently recommended rather than universal screening for all pregnant women [8,10,28,33].

Antibody testing: Antibody testing is an essential diagnostic tool for pregnancy-related thyroid disorders. Anti-thyroid peroxidase (TPOAb) and anti-thyroglobulin antibodies are markers of thyroid autoimmunity, and women with increased serum concentrations of these antibodies tend to have asymptomatic impairment of thyroid function [34,35]. The American Thyroid Association (ATA) recommends that TPOAb status be assessed in all pregnant women with TSH concentrations >2.5 mIU/L [36]. The presence of thyroid autoimmunity in euthyroid women may increase the risk of developing hypothyroidism during pregnancy, and these women should be monitored closely [36]. The diagnosis of subclinical hypothyroidism during pregnancy depends on the recognition of an increased serum TSH level, the analytical method, the antibody status of the mother, ethnicity, iodine nutrition, and even the time of day when the blood is collected [35]. Therefore, antibody testing is an essential component of the diagnostic workup for thyroid disorders during pregnancy, and it can help identify women at risk of developing thyroid dysfunction during pregnancy.

Challenges and Limitations

Interpretation dilemmas: Diagnosing thyroid disorders during pregnancy can present several challenges and limitations, including interpretation dilemmas. Symptoms of thyroid disorders, such as fatigue, weight loss, and palpitations, can be similar to those of normal pregnancy, making it difficult to differentiate between the two [21,27]. Pregnancy is accompanied by hormonal and metabolic changes that can affect thyroid function, complicating the diagnosis of thyroid disorders [10]. In iodine-sufficient regions, the most common causes of thyroid disease during pregnancy are autoimmune thyroiditis and iatrogenic hypothyroidism after treatment for weight gain, decreased exercise capacity, and constipation, which can be confused with common symptoms of pregnancy [6]. The interpretation of thyroid function test results can be challenging due to the wide range of reference values and the influence of various factors, such as maternal antibody status and iodine nutrition [10,36]. Despite these challenges, it is essential to accurately diagnose thyroid disorders during pregnancy to ensure the well-being of both the mother and the baby. Close monitoring and appropriate management of thyroid dysfunction can help minimize the risk of adverse pregnancy outcomes and improve maternal and fetal health [5,10].

Variability in guidelines: Diagnosing thyroid disorders during pregnancy can present challenges and limitations, including guideline variability. The ATA has released guidelines for diagnosing and managing thyroid disease during pregnancy. However, there still needs to be more variation in the interpretation and application of these guidelines [36,37]. Trimester-specific reference intervals for thyroid function tests are essential for accurate diagnosis during pregnancy, as maternal thyroid function can change significantly throughout pregnancy [37]. However, establishing gestation- and laboratory-specific reference intervals, especially for TSH, is challenging due to the variation in maternal thyroid function tests throughout pregnancy [37]. There is ongoing debate on whether to recommend universal thyroid function screening for all pregnant women or targeted screening for high-risk pregnant women [10,36]. The ATA guidelines suggest testing high-risk pregnant women with a personal history of thyroid or other autoimmune disorders or with a family history of thyroid disorders [10,36]. The interpretation of thyroid function test results can be challenging due to the wide range of reference values and the influence of various factors, such as maternal antibody status and iodine nutrition [10,36]. Despite these challenges and limitations, it is essential to accurately diagnose thyroid disorders during pregnancy to ensure the well-being of both the mother and the baby. Close monitoring and appropriate management of thyroid dysfunction can help minimize the risk of adverse pregnancy outcomes and improve maternal and fetal health [4,10].

Management and treatment

Pharmacological Interventions

Thyroid hormone replacement therapy: The management of hypothyroidism during pregnancy typically involves the use of levothyroxine, a synthetic form of the thyroid hormone T4. Levothyroxine is recommended as the preparation of choice for the treatment of hypothyroidism due to its efficacy in resolving the symptoms of hypothyroidism [38,39]. It is usually administered in pill form and is often used to treat an underactive thyroid that is secreting little or no thyroid hormones [40]. The goal of treatment is to reverse clinical progression and correct metabolic derangements, as evidenced by normal blood levels of TSH and free thyroxine (T4) [38]. The treatment goals for hypothyroidism are to provide resolution of the patient's symptoms and hypothyroid signs [41]. Levothyroxine is generally well-tolerated and is the standard of care for treating hypothyroidism, and it is usually taken once a day on an empty stomach [42]. Other treatment alternatives, such as desiccated thyroid extract, are not recommended due to inconsistent hormone levels and potential harm to the fetus [42].

Antithyroid medications: Antithyroid medications, such as methimazole, carbimazole, and propylthiouracil (PTU), are commonly used to treat hyperthyroidism, including Graves' disease, during pregnancy. These medications work by inhibiting the synthesis of thyroid hormones. They have been extensively used for the treatment of various etiologies of hyperthyroidism and are often the preferred therapeutic modality for Graves' disease worldwide [43,44]. However, the use of antithyroid drugs during pregnancy requires careful consideration due to potential risks, such as teratogenicity and liver damage. The primary goals of treatment for hyperthyroidism during pregnancy are to eliminate excess thyroid hormone and minimize the risk of adverse effects on the mother and the fetus [43]. When considering the management of hyperthyroidism in pregnancy, the potential benefits and risks of antithyroid medications should be carefully evaluated, and treatment decisions should be made in consultation with an endocrinologist or other qualified healthcare provider [45,46].

Non-pharmacological Approaches

Dietary considerations: Non-pharmacological approaches can be beneficial to managing thyroid disorders during pregnancy, particularly hyperthyroidism. Some of these approaches include dietary considerations, which can help manage symptoms and improve overall health [47-49]. Consuming foods with anti-inflammatory properties can help alleviate symptoms of hyperthyroidism [49]. Increasing the intake of fiber-rich foods can help improve digestion and overall gut health [49]. Incorporating lean proteins, such as poultry, fish, and low-fat dairy products, can provide high-quality protein and essential nutrients [50]. Healthy fats, such as those found in nuts, seeds, and avocados, can help support hormone production and overall health [50]. Choosing whole grains over refined carbohydrates can provide essential nutrients and fiber, which can help improve digestion [50]. It is essential to consult with a healthcare provider or a registered dietitian to develop a personalized dietary plan that addresses the specific needs and concerns of the pregnant individual. Incorporating these dietary recommendations into managing thyroid disorders during pregnancy can improve the mother's and baby's overall health and well-being.

Lifestyle modifications: Non-pharmacological approaches, such as lifestyle modifications, can be beneficial in managing thyroid disorders during pregnancy. Reducing stress through techniques such as meditation, yoga, or deep breathing can help manage the symptoms of hyperthyroidism [51,52]. Regular exercise can help improve overall health and reduce the symptoms of hyperthyroidism [51,52]. Consuming anti-inflammatory foods, fiber-rich foods, lean proteins, healthy fats, and whole grains can help manage symptoms and improve overall health [51]. Getting enough sleep and maintaining good sleep hygiene can help manage the symptoms of hyperthyroidism [52]. Avoiding triggers such as caffeine, alcohol, and smoking can help manage symptoms of hyperthyroidism [52]. It is essential to consult with a healthcare provider or a registered dietitian to develop a personalized plan that addresses the specific needs and concerns of the pregnant individual. Incorporating these non-pharmacological approaches into managing thyroid disorders during pregnancy can improve the mother's and baby's overall health and well-being.

Educational support: Comprehensive education and support for pregnant individuals with thyroid disorders are integral to effective management. This includes providing guidance on medication adherence, emphasizing the importance of regular check-ups to monitor thyroid function, and promoting lifestyle modifications that support overall health. Patient empowerment through education fosters active participation in their care, enabling individuals to make informed decisions and adopt behaviors that optimize their well-being during pregnancy. A supportive and educational approach contributes to a collaborative healthcare journey, ensuring that pregnant individuals are equipped with the knowledge and tools to navigate the complexities of thyroid disorders and pregnancy [53].

Multidisciplinary Care and Collaboration

Obstetricians and endocrinologists collaboration: The collaboration between obstetricians and endocrinologists is a cornerstone of comprehensive care for pregnant individuals with thyroid disorders. Regular and open communication between these two specialties ensures coordinated management throughout pregnancy. This collaboration is particularly crucial in cases requiring adjustments to medication dosages, considering the dynamic nature of hormonal changes during gestation. Addressing potential complications necessitates a unified approach, where obstetricians and endocrinologists work together to optimize thyroid function, mitigate risks, and prioritize the health of both the expectant mother and the developing fetus. This collaborative model ensures that the complexities of thyroid disorders are managed with precision and attention to obstetric and endocrine considerations [54].

Integration of maternal-fetal medicine specialists: In cases involving complex cases or high-risk pregnancies intertwined with thyroid disorders, integrating maternal-fetal medicine specialists becomes paramount. These specialists bring a nuanced understanding of managing pregnancies complicated by medical conditions, ensuring a holistic approach that prioritizes the well-being of both the mother and the developing fetus. Maternal-fetal medicine specialists contribute their expertise to navigate intricate medical scenarios, offering insights into potential challenges and tailoring management strategies to optimize outcomes. Their involvement enhances the overall quality of care, providing a specialized focus on the unique needs and complexities of pregnancies affected by thyroid disorders [55].

Psychosocial support: Recognizing the psychological impact of managing thyroid disorders during pregnancy, the integration of psychosocial support is imperative. This encompasses various supportive measures, including counseling services and participation in support groups tailored for individuals facing similar challenges. Pregnancy, already a time of heightened emotions, may be further complicated by the stress associated with thyroid disorders. Psychosocial support addresses these emotional aspects, allowing individuals to express concerns, share experiences, and build resilience. By acknowledging and actively addressing the psychosocial dimensions of thyroid disorders during pregnancy, healthcare providers contribute to a more holistic and patient-centered approach, fostering emotional well-being alongside physical health [56].

Impact on fetal development

Fetal Thyroid Function

The impact of maternal thyroid disorders on fetal development is significant, as thyroid hormones play a crucial role in the development of the embryo and fetus [17,57]. Thyroid hormones are essential for developing the central nervous system, and the developing embryo/fetus depends on the maternal supply of thyroid hormones [17,57]. Maternal stress and stress-related biological processes during pregnancy may be important modulators of thyroid function [17]. The development of fetal thyroid function depends on the thyroid gland's embryogenesis, differentiation, and maturation [57]. The fetal thyroid gland does not synthesize thyroid hormone until mid-gestation and severe maternal thyroid deficiency can adversely affect offspring neurodevelopment [17]. Maternal hypothyroxinemia in early pregnancy is a determinant of verbal and nonverbal cognitive functioning in early childhood [58]. Even pregnant women with normal free thyroxine (FT4) concentrations may affect fetal brain development and put children at risk for subsequent cognitive issues [58]. Overt maternal and fetal thyroid disorders are associated with reduced birth weight [59]. Offspring born to pregnant women with isolated hypothyroxinemia have a 6.82-fold increased risk of low birth weight [60]. Thyroid hormones play a vital role in the normal development of bones and muscles, and maternal thyroid dysfunction during early pregnancy has been linked to poor vision development and an increased risk of premature birth [60]. It is essential to closely monitor maternal thyroid function during pregnancy and provide appropriate management to minimize the risk of adverse fetal outcomes.

Neurological and Cognitive Effects

Maternal influences during pregnancy, such as nutrition, infection, and stress, can significantly impact fetal neurodevelopment, potentially leading to intergenerational consequences. An optimal intrauterine environment is crucial for ensuring ideal neurodevelopmental outcomes, and evidence suggests that factors such as maternal nutrition, infection, and stress can affect brain development and outcomes in offspring. Additionally, an adverse intrauterine environment can influence fetal neurodevelopment either through direct effects and maternal signals or indirectly as a consequence of preterm birth, which is independently associated with poor neurodevelopmental outcomes [61].

Furthermore, there is evidence that cognitive functions are affected by the increased levels of sex hormones that occur during pregnancy, mainly during the second and third trimesters. While animal research overwhelmingly supports enhanced memory function during pregnancy, cognitive performance seems to be diminished rather than enhanced in humans. Changes in brain plasticity during pregnancy involve areas implicated in maternal caregiving, reward/motivation, salience/threat detection, emotional regulation, and social cognition, such as the ability to empathize with and infer the baby's mental state [62].

Stress during pregnancy has been shown to have long-lasting effects on the neurodevelopment of the offspring, with altered brain structure and function being associated with prenatal stress. The emotional state of the mother during pregnancy can have long-lasting effects on the psychological development of her child [63,64]. Maternal influences during pregnancy, including nutrition, infection, stress, and hormonal changes, can profoundly impact fetal neurodevelopment and cognitive outcomes, with potentially long-lasting effects on the offspring. It is essential to support pregnant individuals in maintaining a healthy and low-stress environment to promote optimal neurodevelopment and cognitive outcomes for their children.

Long-Term Health Outcomes

Maternal thyroid disorders during pregnancy can have long-term health outcomes for the offspring. Thyroid hormones play a crucial role in fetal development, particularly in the development of the central nervous system [61,65]. Maternal hypothyroxinemia in early pregnancy is a determinant of verbal and nonverbal cognitive functioning in early childhood [66]. Even pregnant women with normal free thyroxine (FT4) concentrations may affect fetal brain development and put children at risk for subsequent cognitive issues [66]. Maternal thyroid dysfunction during pregnancy has been associated with an increased risk of behavioral problems in offspring, including attention-deficit/hyperactivity disorder [64]. Maternal thyroid dysfunction during pregnancy has been linked to an increased risk of metabolic disorders, such as obesity and type 2 diabetes, in offspring [63]. Maternal thyroid dysfunction during pregnancy has been associated with an increased risk of cardiovascular disease in offspring [63]. It is essential to closely monitor maternal thyroid function during pregnancy and provide appropriate management to minimize the risk of adverse fetal outcomes and long-term health consequences for the offspring.

Neonatal thyroid disorders

Transient Hypothyroidism

Transient congenital hypothyroidism (TCH) refers to a temporary deficiency of thyroid hormone identified after birth with low thyroxine (T4) and elevated thyrotropin (TSH), which later recovers to improve thyroxine production, typically in the first few months of infancy [67]. Approximately 17% to 40% of children diagnosed with CH by NBS programs were found to have TCH [67]. The causes of transient CH include prematurity, iodine deficiency, and exposure to antithyroid medications or maternal antithyroid antibodies. The primary determinant of neurodevelopmental outcome is the timing of starting thyroxine. The evidence on whether to treat or not to treat transient hypothyroidism, especially in preterm infants, is still under discussion, and current guidelines are not specific for preterm babies with CH. It is essential to closely monitor and evaluate newborns with transient hypothyroidism to determine the appropriate course of action for their specific condition [67].

Congenital Hypothyroidism

Congenital hypothyroidism (CH) is a condition in which a newborn's thyroid gland does not produce enough thyroid hormones, leading to a deficiency that can cause serious health problems if not treated [68]. It is the most common thyroid disorder in children and can result in symptoms such as slow growth, a lack of activity, and poor performance in school [68]. The most common cause of CH is the failure of the thyroid gland to grow during pregnancy or its location in an abnormal place in the neck, making it work less effectively [68]. TCH is a temporary abnormality of thyroid function in the newborn that later reverts to normal [69]. It can be caused by factors such as iodine deficiency, maternal TSH receptor antibodies, maternal use of antithyroid drugs, dual oxidase 2 mutations, and prematurity [69]. TCH is common in preterm babies, and its recognition can help obviate the risks associated with unnecessary thyroxine treatment [69]. The consequences of untreated CH in infants can be severe, leading to intellectual disability, growth problems, and other health issues [70]. Early diagnosis and treatment ensure normal development and intellectual function in children with CH [70]. It is recommended that the infant be referred to a pediatric endocrinologist for proper care and monitoring [70].

Screening and Early Intervention

NBS for CH is a critical preventive measure that enables early diagnosis and treatment, significantly improving neurodevelopmental outcomes. CH is one of the most common disorders related to mental impairment and growth retardation, occurring in approximately one in 3,000-4,000 infants [71]. Most infants with CH appear normal at birth, underscoring the importance of screening programs for its early detection. The screening typically involves assessing newborns' thyroid and TSH levels. Infants with low T4 and high TSH levels should be considered to have CH and should receive immediate medical attention, including consultation with a pediatric endocrinologist. Early diagnosis and appropriate administration of therapy are crucial in preventing mental retardation and ensuring optimal cognitive outcomes [71]. Screening for CH is recommended to be performed between two and four days of age, and infants discharged from hospitals before 48 hours of life should be tested immediately before discharge. Primary care physicians must ensure that infants with abnormal screening results receive timely follow-up and confirmatory testing. Additionally, families should be provided with comprehensive information about NBS tests, including the potential benefits and limitations, to facilitate informed decision-making [72].

Maternal outcomes and postpartum considerations

Postpartum Thyroiditis

Postpartum thyroiditis is a common condition that affects a significant percentage of women after childbirth. It is characterized by the inflammation of the thyroid gland, which can lead to transient or permanent thyroid dysfunction [73]. The prevalence of postpartum thyroiditis ranges from 5-7% and can vary based on factors such as iodine status and the presence of other autoimmune conditions [74]. The condition typically presents with an initial phase of hyperthyroidism, followed by a hypothyroid phase. In some cases, the thyroid function may return to normal within 12 to 18 months, while in others, permanent hypothyroidism may develop. Postpartum thyroiditis is a strong predictor of future thyroid health, and women who experience this condition should be closely monitored, especially before future pregnancies [73]. Selenium supplementation has been identified as a potential means to prevent postpartum thyroiditis. It is essential for healthcare providers to be aware of this condition and to provide appropriate education and support to women who may be at risk. Regular thyroid tests and close follow-ups are essential for managing postpartum thyroiditis, and most women will experience a return to normal thyroid function within 12 to 18 months after the symptoms start [75].

Long-Term Follow-Up for Maternal Health

Long-term follow-up for maternal health after postpartum thyroiditis is essential, as the condition can have lasting effects on thyroid function. In most cases, thyroid function returns to normal within 12 to 18 months after the onset of symptoms. However, some individuals may not recover from the hypothyroid phase and may develop permanent hypothyroidism [73]. Healthcare providers need to monitor thyroid function in women who have experienced postpartum thyroiditis, especially before future pregnancies, as the condition is a strong predictor of future thyroid health [73]. Regular thyroid tests and close follow-ups are crucial for managing postpartum thyroiditis, and most women will experience a return to normal thyroid function within 12 to 18 months after the symptoms start [73]. This follow-up is essential to ensure the mother's well-being and address any potential long-term effects of postpartum thyroiditis on thyroid function and overall health [73,74].

Breastfeeding Considerations

Breastfeeding considerations for mothers with thyroid issues are crucial, as these conditions can impact milk production and the baby's health. Hypothyroidism can lead to delayed or insufficient milk production, and studies suggest it may negatively affect oxytocin and prolactin concentrations [76]. PTU is the preferred anti-thyroid drug for breastfeeding mothers, as it is excreted in small amounts into breast milk and does not significantly impact the baby's thyroid function. Methimazole is an accepted option, but the baby should be monitored frequently. Postpartum thyroid dysfunction, including postpartum thyroiditis, can cause difficulty with milk supply and removal, and mothers may experience changes in their thyroid levels during pregnancy and childbirth, necessitating frequent testing [77]. It is recommended that breastfeeding mothers receive regular follow-ups with a physician and screening for thyroid function to support their breastfeeding journey. It is essential to balance the management of thyroid issues during breastfeeding to ensure the well-being of both the mother and the baby [78].

## Conclusions

This comprehensive review has illuminated the intricate dynamics of thyroid disorders during pregnancy, spanning from conception to postpartum considerations. Key findings underscore the profound impact of maternal thyroid health on fetal and neonatal development, emphasizing the critical need for timely screening and targeted interventions. The evolving landscape of research and technology suggests a shift toward personalized approaches in clinical practice, necessitating integrated care models and the incorporation of telehealth platforms. Postpartum considerations, such as postpartum thyroiditis, further underscore the importance of ongoing monitoring and intervention for maternal well-being. The implications for clinical practice extend to healthcare provider education, public awareness campaigns, and policy advocacy for standardized screening guidelines. As a call to action, there is a pressing need for increased research funding to advance our understanding and improve outcomes. By fostering awareness, education, and collaborative efforts, we can navigate the complexities of thyroid disorders during pregnancy and ensure a healthier start for both mothers and their infants.
